# Supplementary material for: Intrinsic neuronal dynamics predict distinct functional roles during working memory
Source: Nat Commun. 2018 Aug 29;9:3499. doi: 10.1038/s41467-018-05961-4 (PMC6115413; doi:10.1038/s41467-018-05961-4)
Supplement: Supplementary file 1 — Supplementary information [file 41467_2018_5961_MOESM1_ESM.pdf]

Supplementary information

Title: “Intrinsic neuronal dynamics predict distinct functional roles during working memory”

Authors: Wasmuht et al.

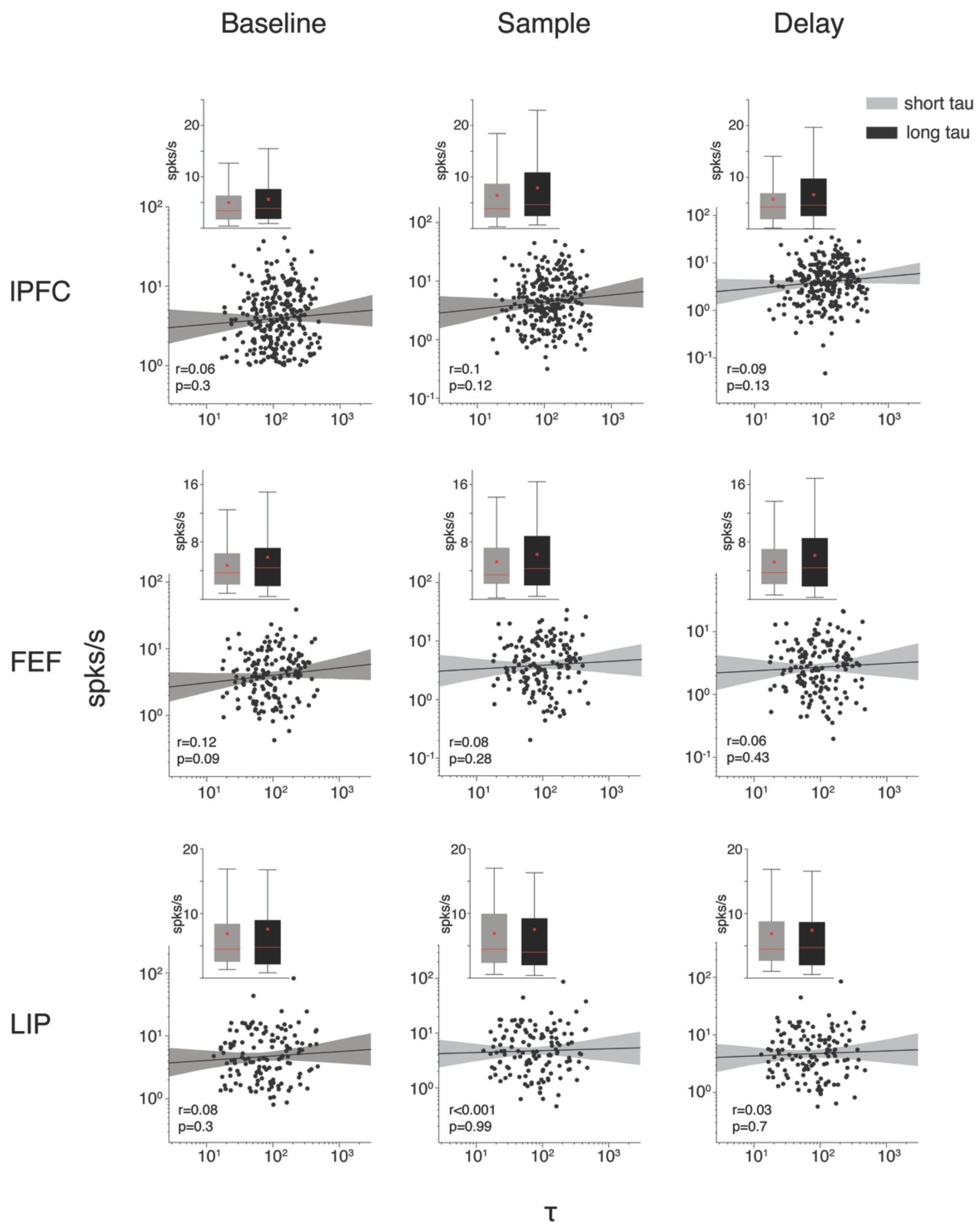

Supplementary Figure 1: Relationship between tau and firing rate. Log scaled scatter plots show tau (x-axis) against trial averaged firing rate (y-axis) for each task epoch (columns) and each brain region (rows). Every dot represents a neuron. (lPFC: n=265; FEF: n=168; LIP: n=136). Spearman rank correlation coefficients and associated p-values are displayed in the lower left corners. Note that none of the correlation coefficients was significant. Black solid lines depict linear fits to the log transformed data. Shaded area depicts 95% confidence intervals of the linear fits. Insets depict boxplots of epoch specific neuronal firing rates grouped according to associated short and long tau values (as determined by a median split). Red lines indicate median firing rate. Red squares indicate mean firing rate. Box edges represent top and bottom quartiles with whiskers indicating (1.5*interquartile range) in both directions. Outliers are not plotted. We performed a Wilcoxon rank sum test to evaluate whether the firing rates for short and long tau cells were drawn from the same distribution but found no evidence defying the null hypothesis: lPFC (Wilcoxon rank sum test, Baseline: p=0.21; Sample: p=0.13; Delay: p=0.75), FEF (Baseline: p=0.25; Sample: p=0.56; Delay: p=0.75), LIP (Baseline: p=0.87; Sample: p=0.53; Delay: p=0.7).

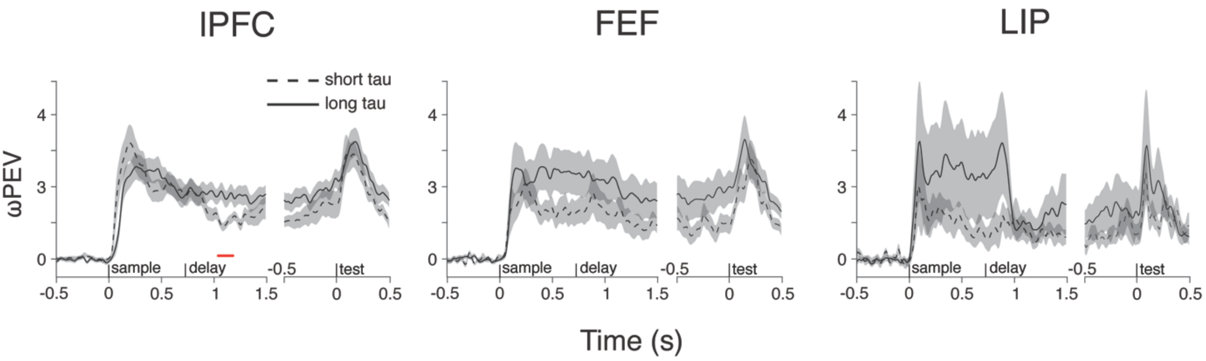

Supplementary Figure 2: Item information and intrinsic timescales for cells showing a significant ωPEV at any time point during the trial. Same conventions as in Fig. 2 hold true. Red line indicates a significant difference (cluster based permutation test, p=0.04). lPFC (n=203); FEF (n=121); LIP (n=53).

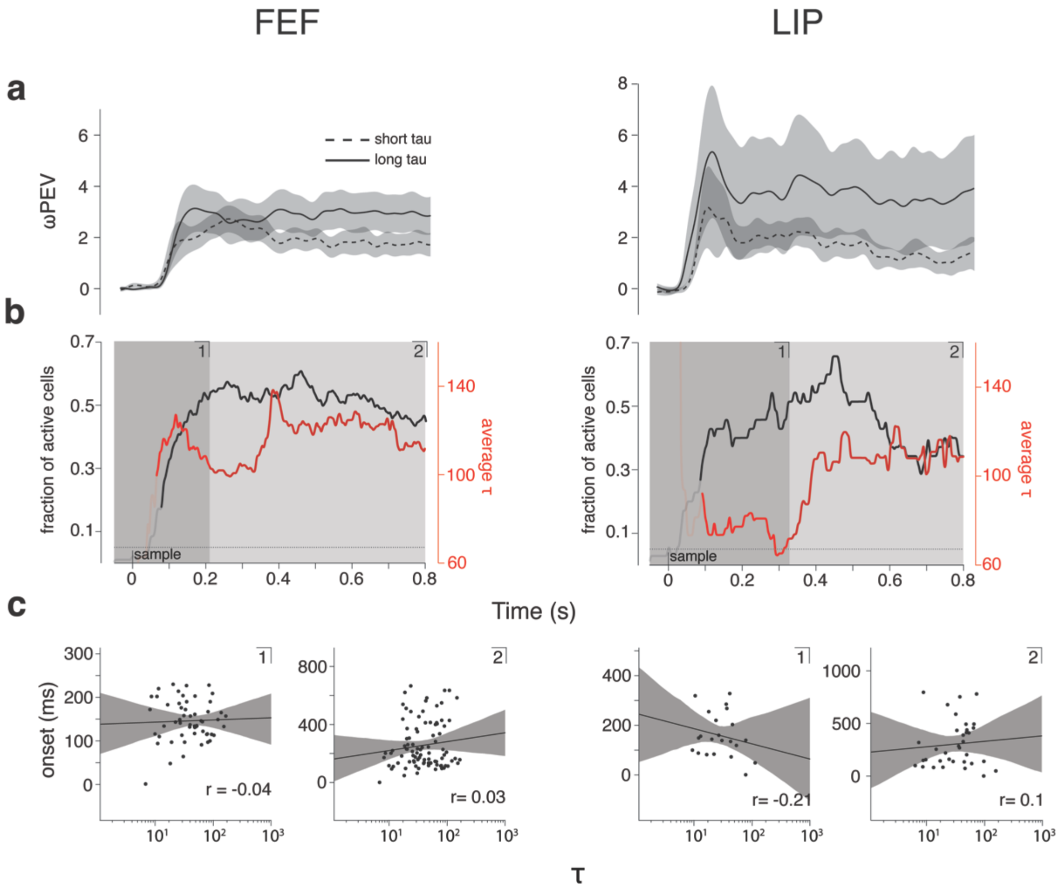

Supplementary Figure 3: **Evaluation of sample period onset times in relation to intrinsic timescales for FEF and LIP.** This figure shows the same plots as Fig. 3, for FEF (left column) and LIP (right column). **a)** Average ωPEV for cells with significant ωPEV at any time point during the sample period. **b)** Average tau for cells with significant ωPEV at time point on x-axis (red line) and fraction of cells with significant ωPEV at time point on x-axis out of all cells showing a significant ωPEV during the sample period. **c)** Scatter plots of encoding onsets (first point of significant ωPEV) versus tau for time periods 1 and 2. FEF: Time period **1** (Spearman rank correlation, r=-0.04, p=0.76, n=56); Time period **2** (r=0.04, p=0.71, n=96). LIP: Time Period **1**: (r=-0.21, p=0.34, n=22); Time Period **2**: (r=0.1, p=0.57, n=35).

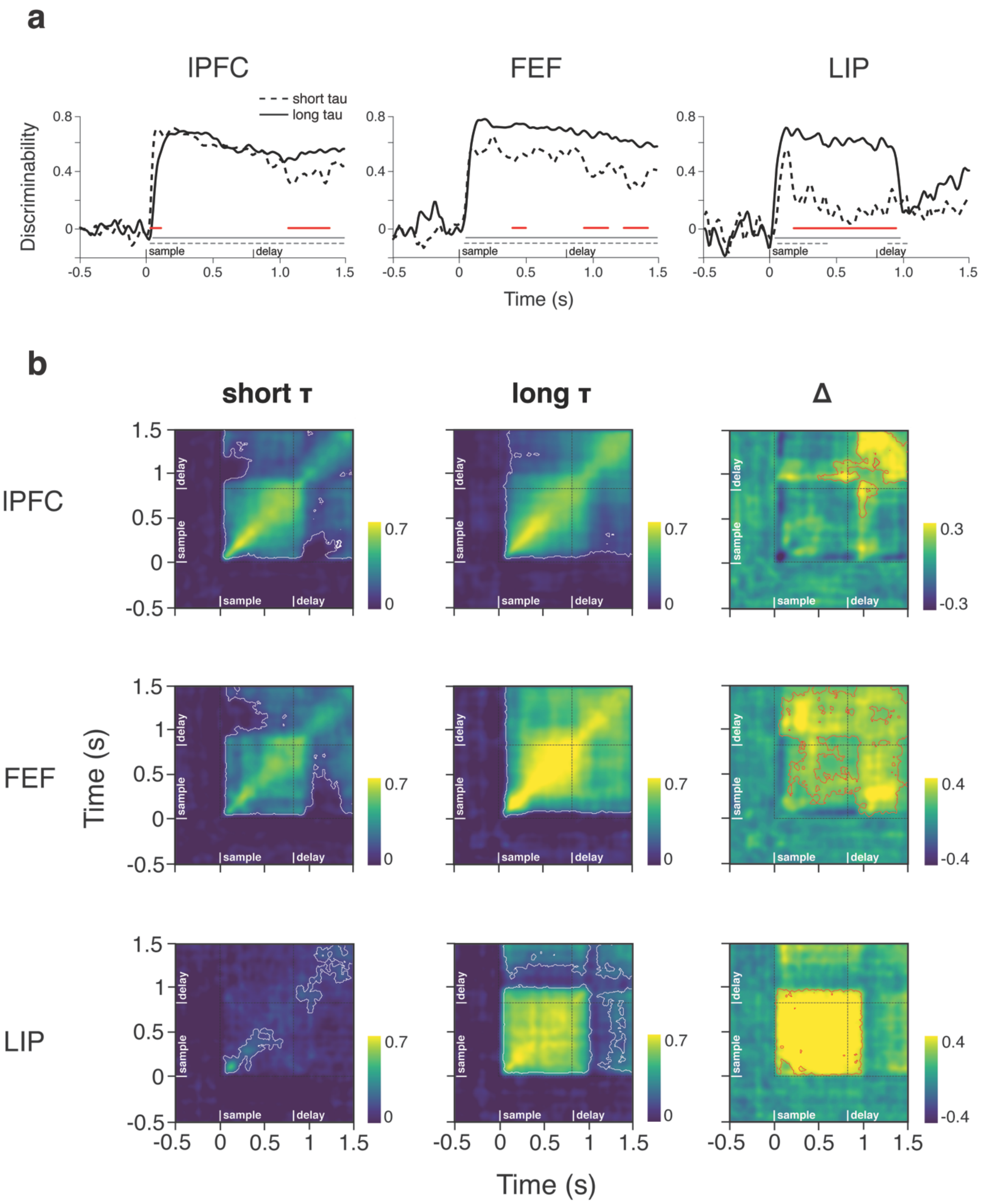

Supplementary Figure 4: Within-time and cross-temporal decoding for location information. Same as Fig. 4 but for location information only (see Methods). **a)** Within time decoding. **b)** Across time decoding. White contours: cluster based permutation test, p<0.001; Red contours: p<0.01. Color scale depicts decoding discriminability.

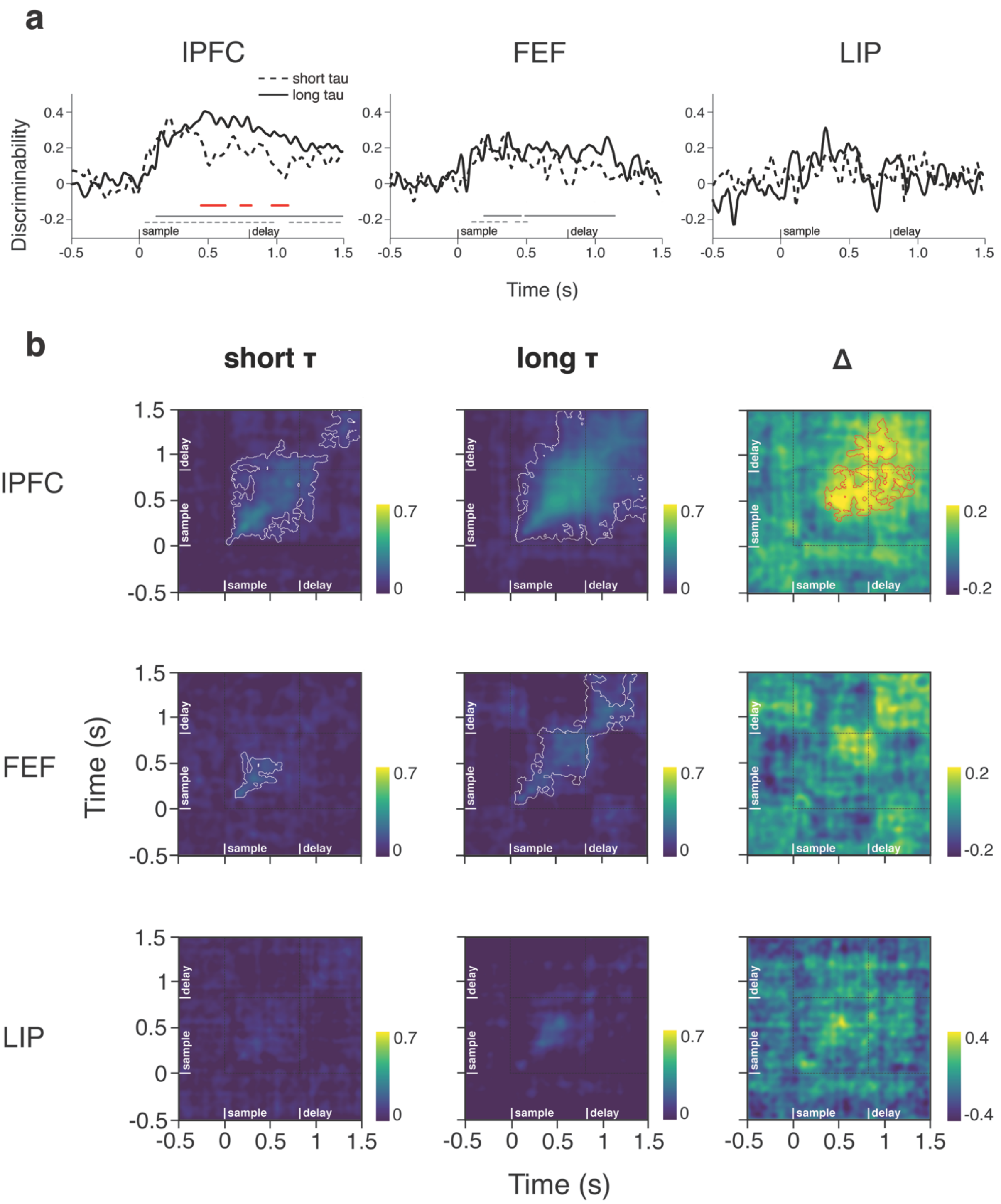

Supplementary Figure 5: Within-time and cross-temporal decoding for color information. Same as Fig. 4 but for color information only (see Methods). **a)** Within time decoding. **b)** Across time decoding. White contours: cluster based permutation test, p<0.001; Red contours: p<0.01. Color scale depicts decoding discriminability.

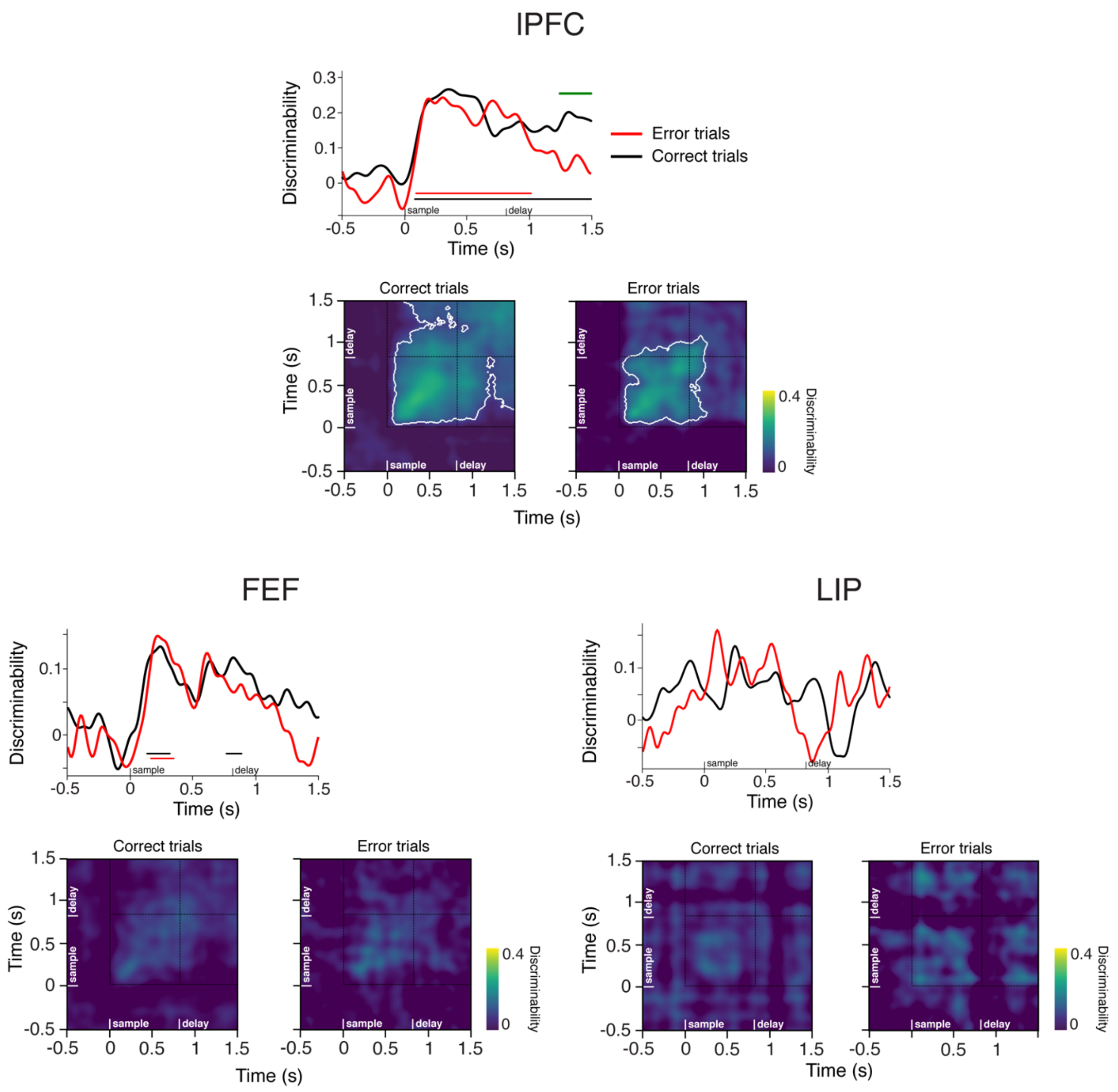

Supplementary Figure 6: Correct versus error trial target decoding. Within and across time target decoding within correct and error trials for each brain region. Within time target discriminability is depicted in the upper panels for each region. Black and red lines indicate discriminability for correct and error trial, respectively. The correspondingly colored horizontal lines indicate significant discriminability (cluster based permutation test, $p<0.05$). The green line indicates a significant difference between correct and error trials (cluster based permutation test, $p=0.035$). The lower two panels indicate the across time target decoding for each brain region. The discriminability score is color coded as indicated by the color bars. White contours indicate significant cross-temporal discriminability (cluster based permutation test, $p<0.05$). Black dashed lines distinguish task periods. lPFC (n=60); FEF (n=50); LIP (n=35).

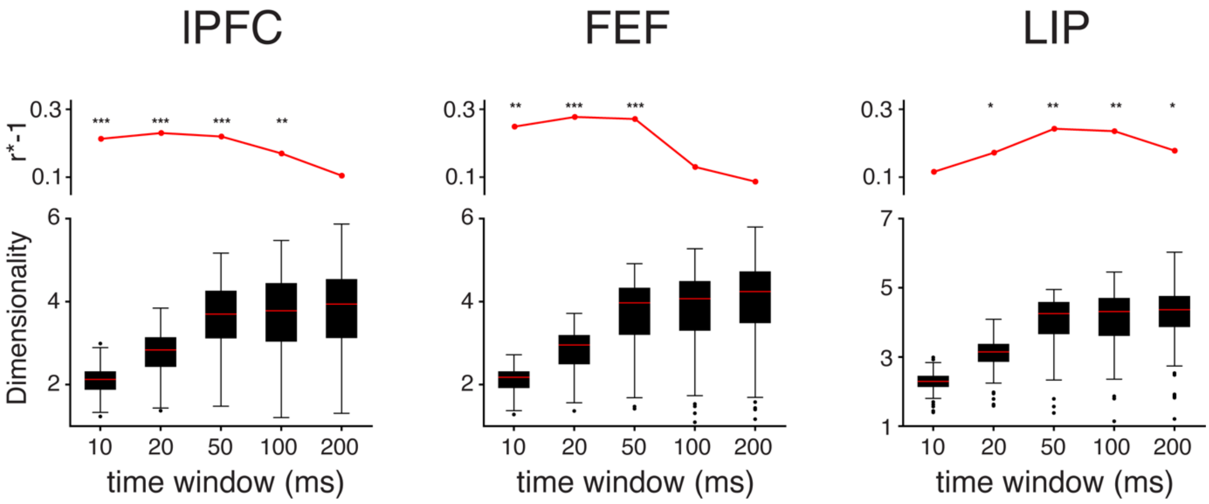

Supplementary Figure 7: Effective temporal dimensionality estimated over different time windows and relationship with intrinsic timescales. Lower panels: Boxplots show N_{eff} estimated over differing time-windows (10ms*10, 20ms*10, 50ms*10, 100ms*10 and 200ms*10). Red lines denote median N_{eff} for each time-window over all cells, black boxes mark the upper and lower quartiles. Whiskers denote 15* interquartile range. Dots represent outliers. Upper panels: Each dot represents the Spearman rank correlation coefficient of N_{eff} and τ values, for each of the time window N_{eff} is estimated from. * represents p<0.05; ** represents p<0.01; *** represents p<0.001. lPFC (n=264); FEF (n=167); LIP (n=134).

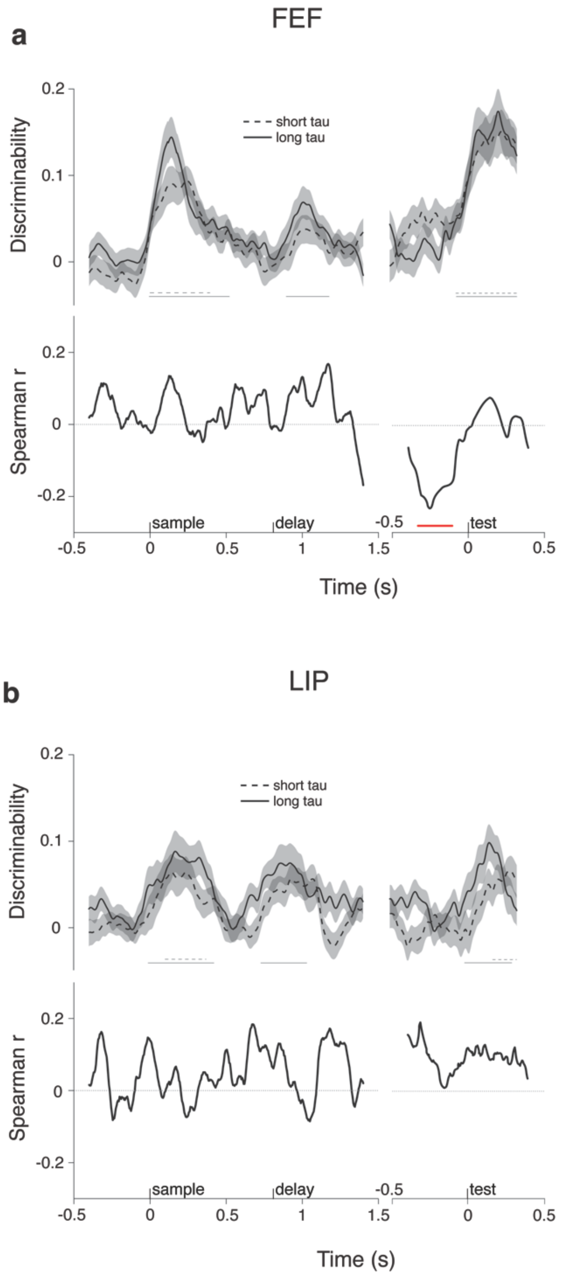

Supplementary Figure 8: Temporal discriminability in FEF and LIP. Same conventions as in Fig. 6. **a)** FEF; **b)** LIP. Red line indicated a significant difference (cluster based permutation test, p<0.05)

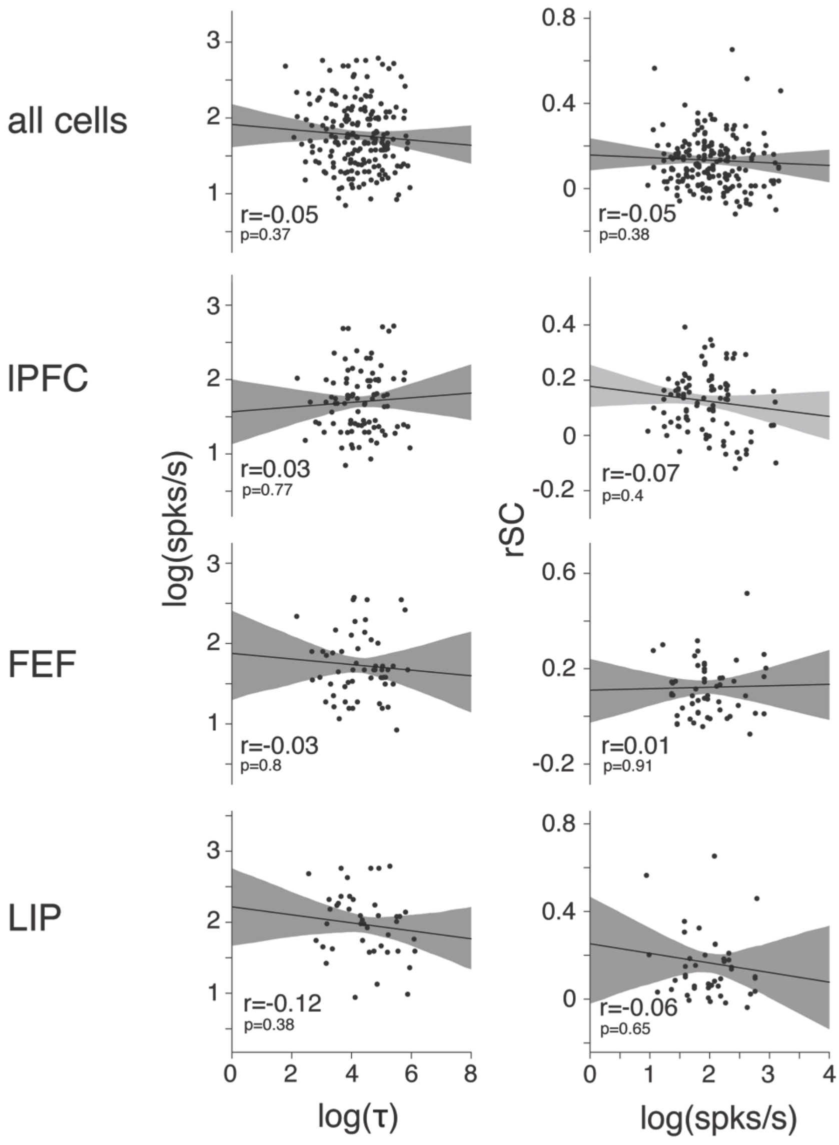

Supplementary Figure 9: Spike count correlations and intrinsic timescales. The first column shows scatter plots for the log transformed fixation period firing rate in spks*s^{-1} on the y-axis, versus the log transformed τ values on the x-axis. The r value within panels depicts the Spearman rank correlation coefficient for the two variables. Black solid lines show linear regression lines. Shaded region depicts 95% confidence interval. The second column shows scatter plots the log transformed fixation period firing rate (spks*s^{-1}) on the x-axis and rSC on the y-axis. The r value within panels depicts the Spearman rank correlation coefficient and associated p-value, between the two variables. All cells (n=255); lPFC (n=126); FEF (n=74); LIP (n=55).
